# Spermatological characteristics of Pleurogenidae (Digenea) inferred from the ultrastructural study of *Pleurogenes claviger*, *Pleurogenoides medians* and *Prosotocus confusus*

**DOI:** 10.1051/parasite/2013028

**Published:** 2013-08-29

**Authors:** Jordi Miquel, Daniel Vilavella, Zdzisław Świderski, Vladimir V. Shimalov, Jordi Torres

**Affiliations:** 1 Laboratori de Parasitologia, Departament de Microbiologia i Parasitologia Sanitàries, Facultat de Farmàcia, Universitat de Barcelona Av. Joan XXIII, sn 08028 Barcelona Spain; 2 Institut de Recerca de la Biodiversitat, Facultat de Biologia, Universitat de Barcelona Av. Diagonal 645 08028 Barcelona Spain; 3 W. Stefański Institute of Parasitology, Polish Academy of Sciences 51/55 Twarda Street 00-818 Warsaw Poland; 4 Department of General Biology and Parasitology, Warsaw Medical University 5 Chałubińskiego Street 02-004 Warsaw Poland; 5 Brest State University 224665 Brest Belarus

**Keywords:** *Pleurogenes claviger*, *Pleurogenoides medians*, *Prosotocus confusus*, Spermatozoon ultrastructure, Comparative spermatology

## Abstract

The present work constitutes the first ultrastructural analysis of the spermatozoon in the Pleurogenidae, with the study of three species belonging to three of the 16 genera included in this family, namely *Pleurogenes claviger*, *Pleurogenoides medians* and *Prosotocus confusus*. The mature spermatozoa of these pleurogenids present two axonemes of the 9+“1” trepaxonematan pattern, a nucleus, two mitochondria, two bundles of parallel cortical microtubules, external ornamentation, spine-like bodies and granules of glycogen. The organization of these characters in the sperm cell is similar in the three species. Thus, the anterior spermatozoon extremity is filiform and a continuous and submembranous layer of parallel cortical microtubules surrounds the axonemes at their anterior end. The posterior spermatozoon extremity exhibits the second axoneme and corresponds to the Cryptogonimidean type of Quilichini *et al.* (2010). Slight differences were noted between the spermatozoon of *P. confusus* and those of the two remaining species in the location of mitochondria.

## Abstract (fr)

Ce travail constitue la première analyse ultrastructurale du spermatozoïde chez les Pleurogenidae, avec l’étude de trois espèces appartenant à trois des 16 genres inclus dans cette famille : *Pleurogenes claviger*, *Pleurogenoides medians* et *Prosotocus confusus*. Le spermatozoïde mûr de ces Pleurogenidae présente deux axonèmes du type 9+‘1’ caractéristique des Trepaxonemata, un noyau, deux mitochondries, deux champs de microtubules corticaux parallèles, des ornementations externes, des corps épineux et des granules de glycogène. L’organisation de ces caractères dans le spermatozoïde est similaire dans les trois espèces. Ainsi, l’extrémité antérieure du spermatozoïde est filiforme et les deux axonèmes sont initialement entourés par une couche continue et sous-membranaire de microtubules corticaux parallèles. L’extrémité postérieure contient le deuxième axonème et cette extrémité correspond au type Cryptogonimidae de Quilichini *et al*. (2010). Des légères différences existent entre le spermatozoïde de *P. confusus* et les deux autres espèces pour la position des mitochondries.

## Introduction

Ultrastructural characteristics of spermatozoa were proved to be valuable tools for the analysis of phylogenetic relationships within the Platyhelminthes, particularly for the Eucestoda but also for the Monogenea [3, 22, 31–36, 46, 83]. It is generally accepted that this source of characters contributes to the establishment of a more robust phylogeny when they are combined with morphological, biological and/or molecular data [28, 29, 35, 46, 66, 89]. Considering digeneans, during the last years there was an important increase of ultrastructural studies on the spermatozoon, as reviewed by Bakhoum [5]. However, numerous groups such as the Pleurogenidae have been neglected and no data were available until now.

Within the Digenea, the superfamily Microphalloidea includes 18 families [17] and, to our knowledge, there are ultrastructural studies on the spermatozoon of only six species, namely *Pronoprymna ventricosa* (Faustulidae), *Maritrema linguilla* and *Microphallus primas* (Microphallidae), *Postorchigenes gymnesicus* (Phaneropsolidae), *Mediogonimus jourdanei* (Prosthogonimidae) and *Diphterostomum brusinae* (Zoogonidae) [8, 18, 25, 26, 49, 75]. Nevertheless, in the case of microphallids the studies on *M. linguilla* and *M. primas* [18, 26] are poorly illustrated and contain numerous misinterpretations and consequently, the ultrastructural organization of the spermatozoon is not clear for these species.

The family Pleurogenidae includes species parasitizing mainly amphibians, but some species have also been reported in reptiles and sometimes in fish and mammals, probably as accidental infections [51]. The present study includes data about three (*Pleurogenes*, *Pleurogenoides* and *Prosotocus*) of the 16 genera included in the Pleurogenidae [51]. Thus, we present the first spermatological data of this family, with the study of the ultrastructural organization of the mature spermatozoon of *Pleurogenes claviger*, *Pleurogenoides medians* and *Prosotocus confusus*.

## Materials and methods

Specimens of *Pleurogenes claviger* (Rudolphi, 1819) [79] were collected from the intestine of a naturally infected *Hyla arborea* (Amphibia, Hylidae) whereas *Pleurogenoides medians* (Olsson, 1876) [67] and *Prosotocus confusus* (Looss, 1894) [50] were collected from the intestine of naturally infected *Rana lessonae* (Amphibia, Ranidae). Hosts were captured in April 2008 in the Bugskiy landscape reserve (Southwest Belarus) by V.V. Shimalov according to the Belarusian laws.

After dissection, live digeneans were routinely processed for TEM examination. Therefore, they were fixed in cold (4 °C) 2.5% glutaraldehyde in a 0.1 M sodium cacodylate buffer at pH 7.4 for a minimum of 2 h, rinsed in a 0.1 M sodium cacodylate buffer at pH 7.4, postfixed in cold (4 °C) 1% osmium tetroxide (OsO_4_) with 0.9% potassium ferricyanide [K_3_Fe(CN)_6_] in the same buffer for 1 h, rinsed in milliQ water, dehydrated in an ethanol series and propylene oxide, embedded in Spurr’s resin and finally polymerized at 60 °C for 72 h. Ultrathin sections (50–60 nm thick) were obtained using a Reichert-Jung Ultracut E ultramicrotome, placed on copper grids and double-stained with uranyl acetate and lead citrate according to Reynolds [78] methodology. Ultrathin sections were examined using a JEOL 1010 TEM operated at an accelerating voltage of 80 kV.

The Thiéry [86] technique was used to locate glycogen. Gold grids were treated in periodic acid, thiocarbohydrazide and silver proteinate (PA-TCH-SP) as follows: 30 min in 10% PA, rinsed in milliQ water, 24 h in TCH, rinsed in acetic solutions and milliQ water, 30 min in 1% SP in the dark and rinsed in milliQ water.

## Results

**Figure 1.**
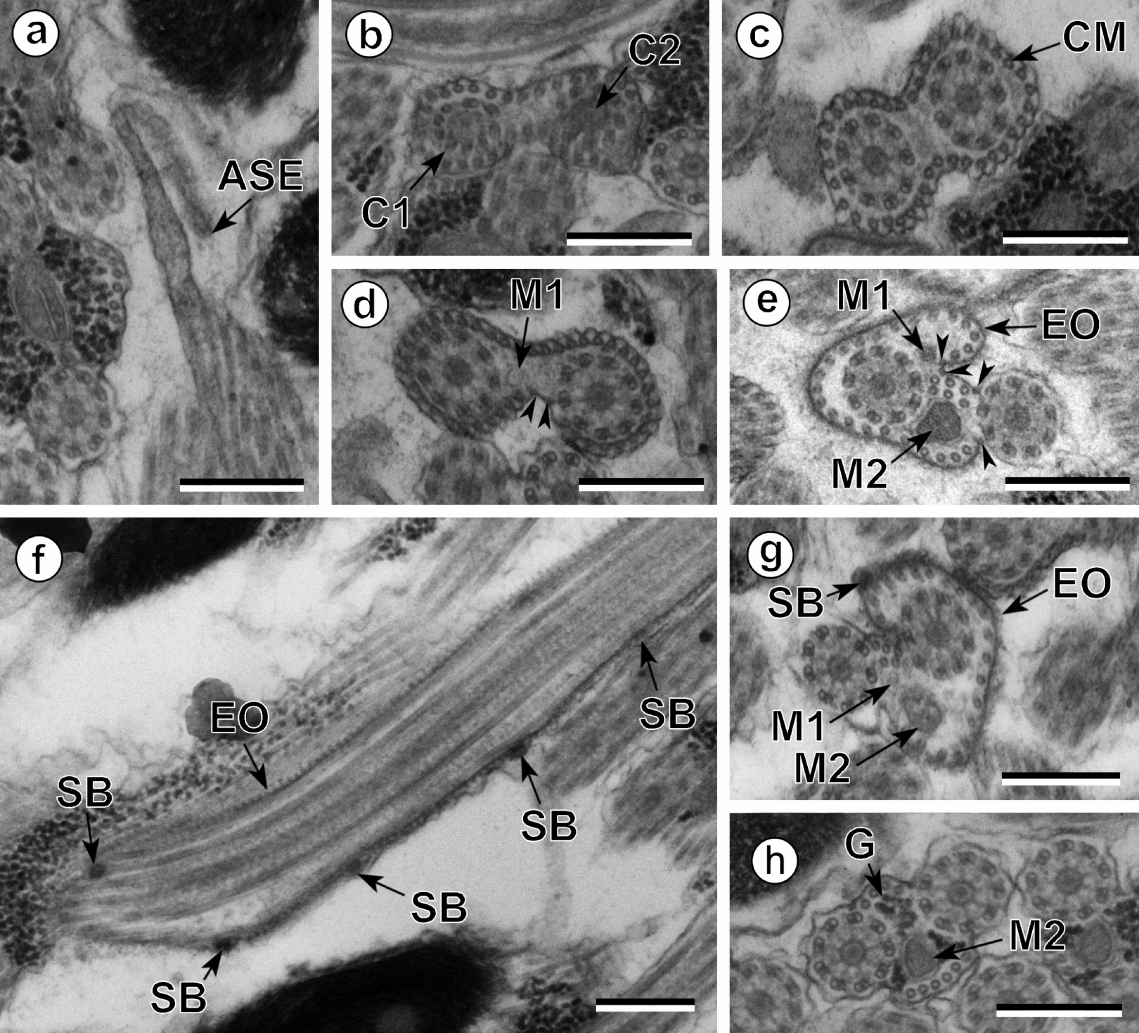
Spermatozoon of *Pleurogenes claviger*, anterior and middle parts (Regions I and II). (a) Longitudinal section of the anterior spermatozoon extremity (ASE). (b, c) Consecutive sections of the anterior area of the sperm cell showing the two centrioles (C1 and C2) and the presence of the complete submembranous layer of parallel cortical microtubules (CM). (d) Cross-section showing the appearance of the first mitochondrion (M1) and two attachment zones (arrowheads). (e–g) Longitudinal and cross-sections of the ornamented area of the spermatozoon. Note the presence of external ornamentation of the plasma membrane (EO), spine-like bodies (SB) and the second mitochondrion (M2). In this area, the cortical microtubules are organized into two fields separated by four attachment zones (arrowheads). M1, first mitochondrion. (h) Cross-section of the middle part of the spermatozoon (Region II) showing the appearance of granules of glycogen (G) and the presence of only the second mitochondrion (M2). Scales in μm: 0.3.

**Figure 2.**
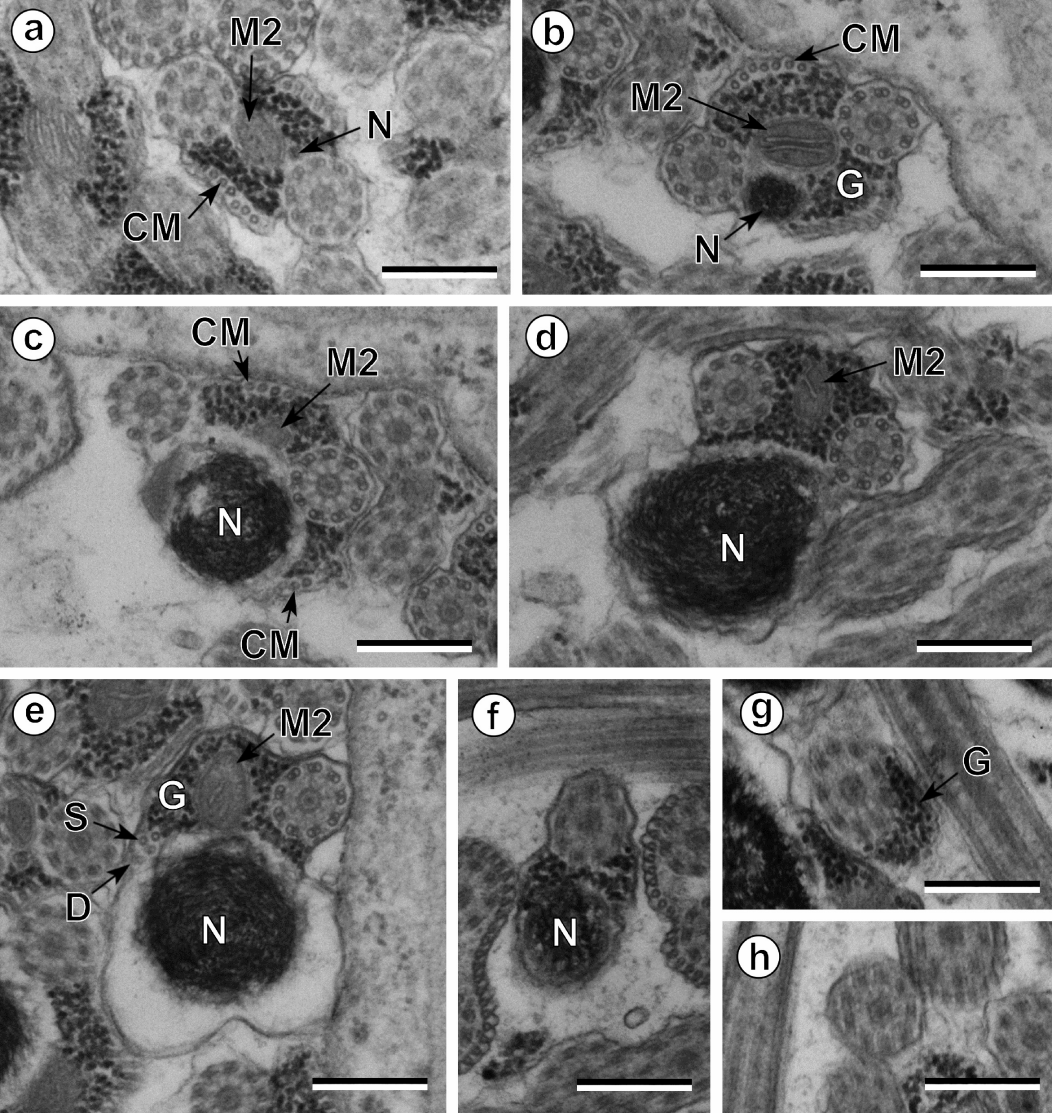
Spermatozoon of *Pleurogenes claviger*, posterior or nuclear part (Region III); all cross-sections. (a–c) Consecutive sections of the nuclear or posterior area of the sperm cell showing the appearance of the nucleus (N) and its progressively increasing size. CM, cortical microtubules; G, granules of glycogen; M2, second mitochondrion. (d) Absence of cortical microtubules. M2, second mitochondrion; N, nucleus. (e) Level of the disorganization of the first axoneme into doublets (D) and singlets (S). G, granules of glycogen; M2, second mitochondrion; N, nucleus. (f) Disappearance of the second mitochondrion and the reduction of the nucleus’ section (N). (g, h) Posterior spermatozoon extremity with and without granules of glycogen (G). Scales in μm: 0.3.

**Figure 3.**
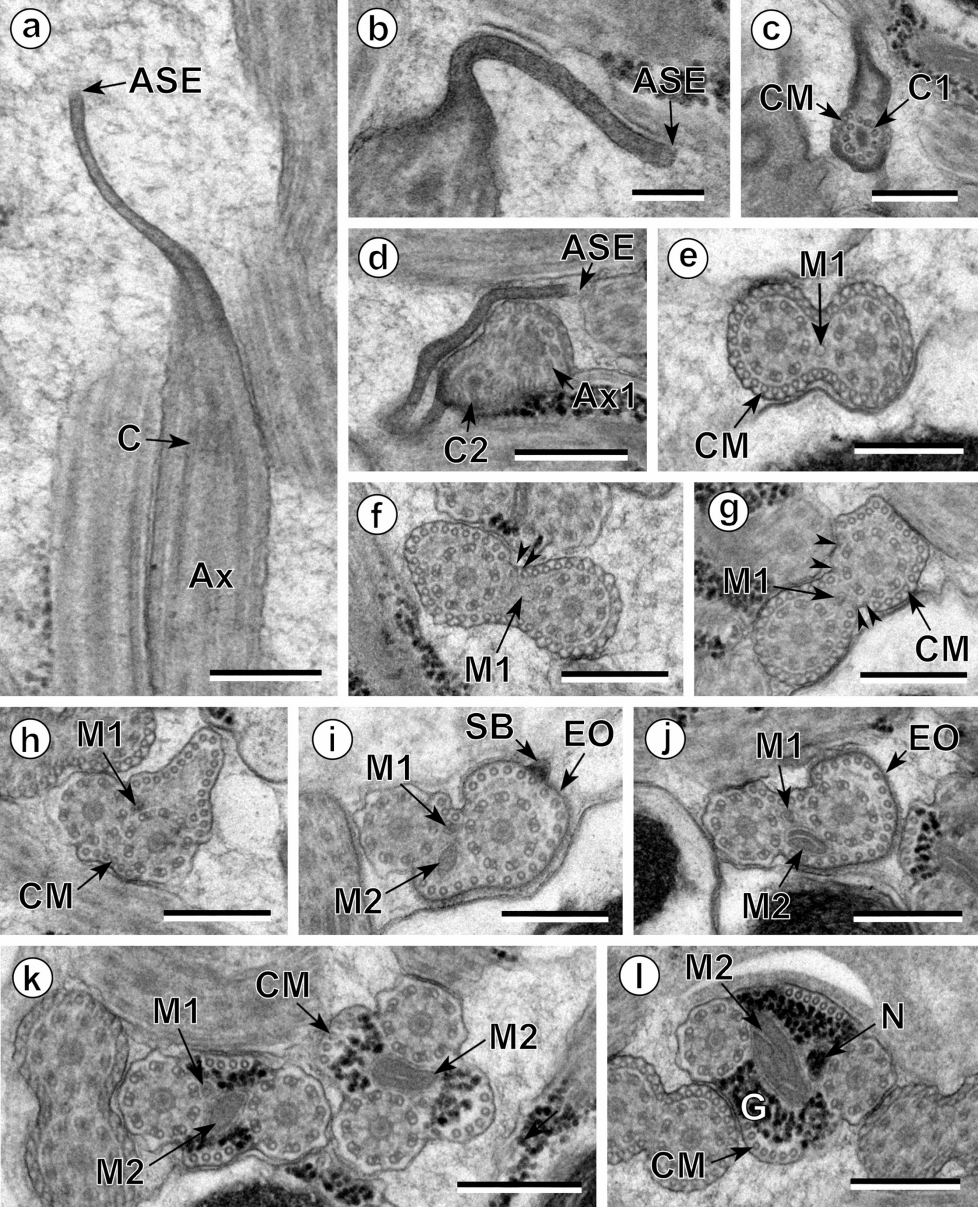
Spermatozoon of *Pleurogenoides medians*. (a, b) longitudinal sections; all others, cross-sections. (a) Anterior spermatozoon extremity (ASE). Ax, axoneme; C, centriole. (b) Detail of the anterior spermatozoon extremity (ASE). (c, d) Consecutive sections showing the appearance of the centrioles (C1 and C2) and cortical microtubules (CM). (e–h) Consecutive sections of the anterior area of the spermatozoon showing the appearance of the first mitochondrion (M1) and the two and four attachment zones (arrowheads). Thus, parallel cortical microtubules (CM) transform from a submembranous continuous layer into two fields. (i, j) Ornamented area. Note the presence of external ornamentation of the plasma membrane (EO), spine-like bodies (SB) and the second mitochondrion (M2). M1, first mitochondrion. (k) Middle part of the sperm cell (or Region II) showing the stopping of the first mitochondrion (M1). CM, cortical microtubules; M2, second mitochondrion. (l) Anterior part of the nuclear region. CM, cortical microtubules; G, granules of glycogen; M2, second mitochondrion; N, nucleus. Scales in μm: (a, c–l), 0.3; (b), 0.1.

**Figure 4.**
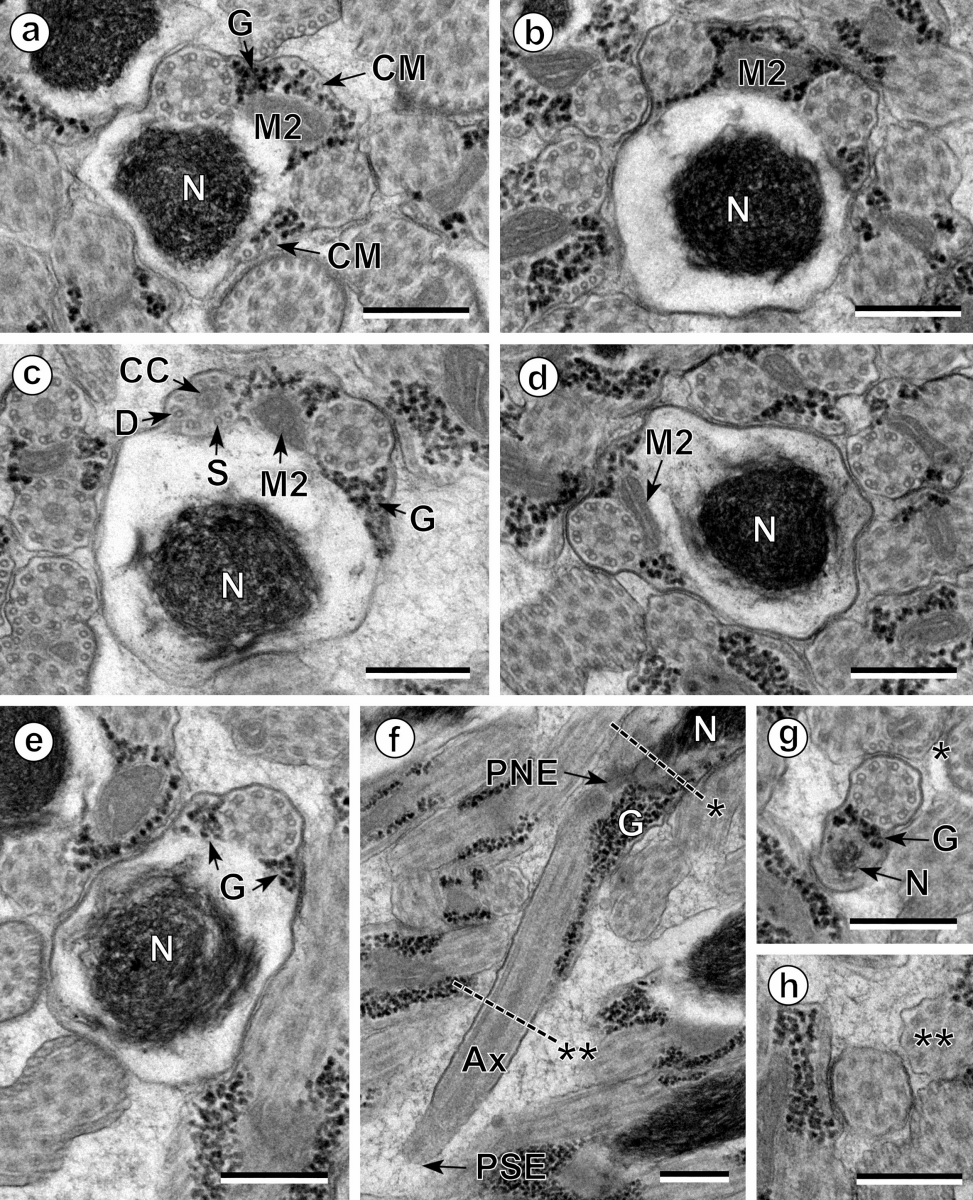
Spermatozoon of *Pleurogenoides medians*, posterior or nuclear part (Region III); all cross-sections except (f). (a, b) End of the two bundles of cortical microtubules (CM); G, granules of glycogen; M2, second mitochondrion; N, nucleus. (c) Disorganization of the first axoneme resulting into the central core (CC), doublets (D) and singlets (S). G, granules of glycogen; M2, second mitochondrion; N, nucleus. (d, e) Disappearance of the second mitochondrion (M2). G, granules of glycogen; N, nucleus. (f) Posterior spermatozoon extremity (PSE). Note the posterior nuclear extremity (PNE). Cross-sections of levels * and ** are shown in (g) and (h). Ax, axoneme; G, granules of glycogen; N, nucleus. (g, h) Sections similar to level of * and **, respectively, in (f). G, granules of glycogen; N, nucleus. Scales in μm: 0.3.

**Figure 5.**
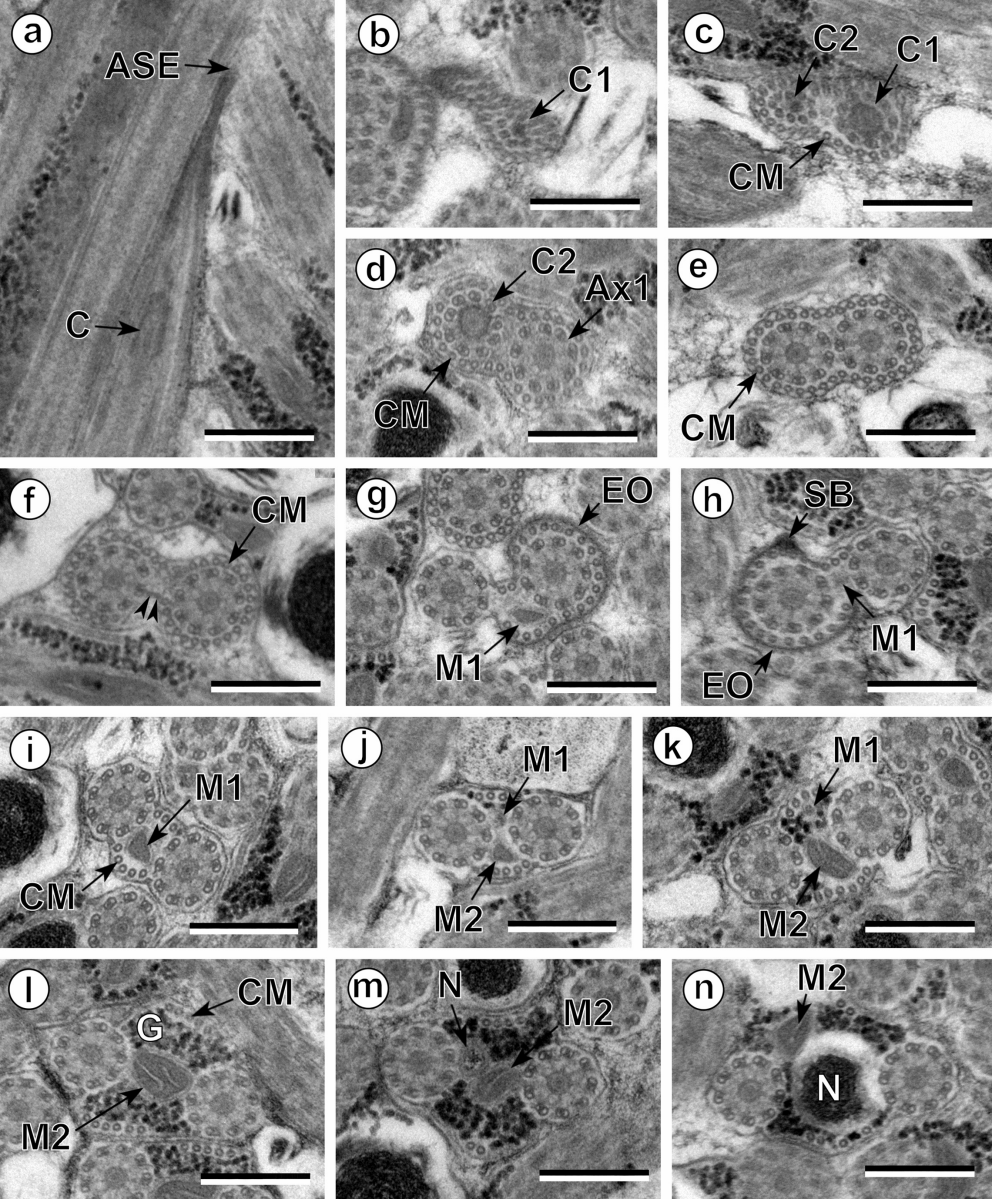
Spermatozoon of *Prosotocus confusus*; all cross-sections except (a). (a) Longitudinal section of the anterior spermatozoon extremity (ASE). C, centriole. (b–e) Consecutive sections of the anterior area of the spermatozoon containing a complete submembranous layer of cortical microtubules (CM). Ax1, first axoneme; C1, centriole of the first axoneme; C2, centriole of the second axoneme. (f) Presence of two attachment zones (arrowheads). CM, cortical microtubules. (g, h) Ornamented area of the sperm cell characterized by the presence of external ornamentation of the plasma membrane (EO), spine-like bodies (SB) and the first mitochondrion (M1). (i–l) Middle area of the spermatozoon (Region II) showing one or two mitochondria. CM, cortical microtubules; G, granules of glycogen; M1, first mitochondrion; M2, second mitochondrion. (m, n) Anterior part of the nuclear region of the spermatozoon (Region III). M2, second mitochondrion; N, nucleus. Scales in μm: 0.3.

**Figure 6.**
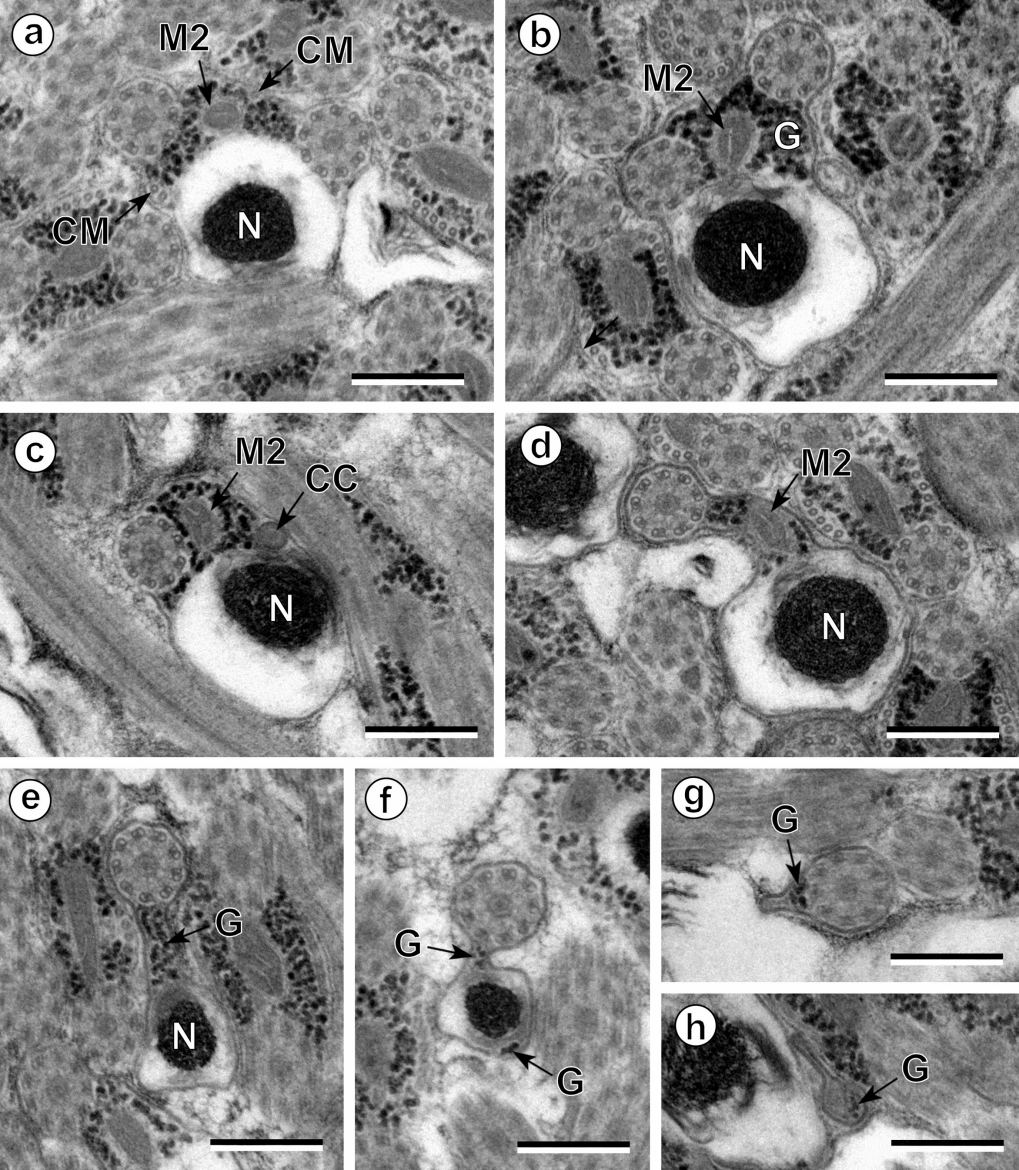
Spermatozoon of *Prosotocus confusus*, posterior or nuclear part (Region III); all cross-sections. (a, b) Disappearance of cortical microtubules (CM); G, granules of glycogen; M2, second mitochondrion; N, nucleus. (c) Level of disorganization of the first axoneme; CC, central core; M2, second mitochondrion; N, nucleus. (d–f) Consecutive sections showing the disappearance of the second mitochondrion (M2) and the progressive reduction of the size of nucleus (N). G, granules of glycogen. (g, h) Level of the posterior spermatozoon tip. G, granules of glycogen. Scales in μm: 0.3.

**Figure 7.**
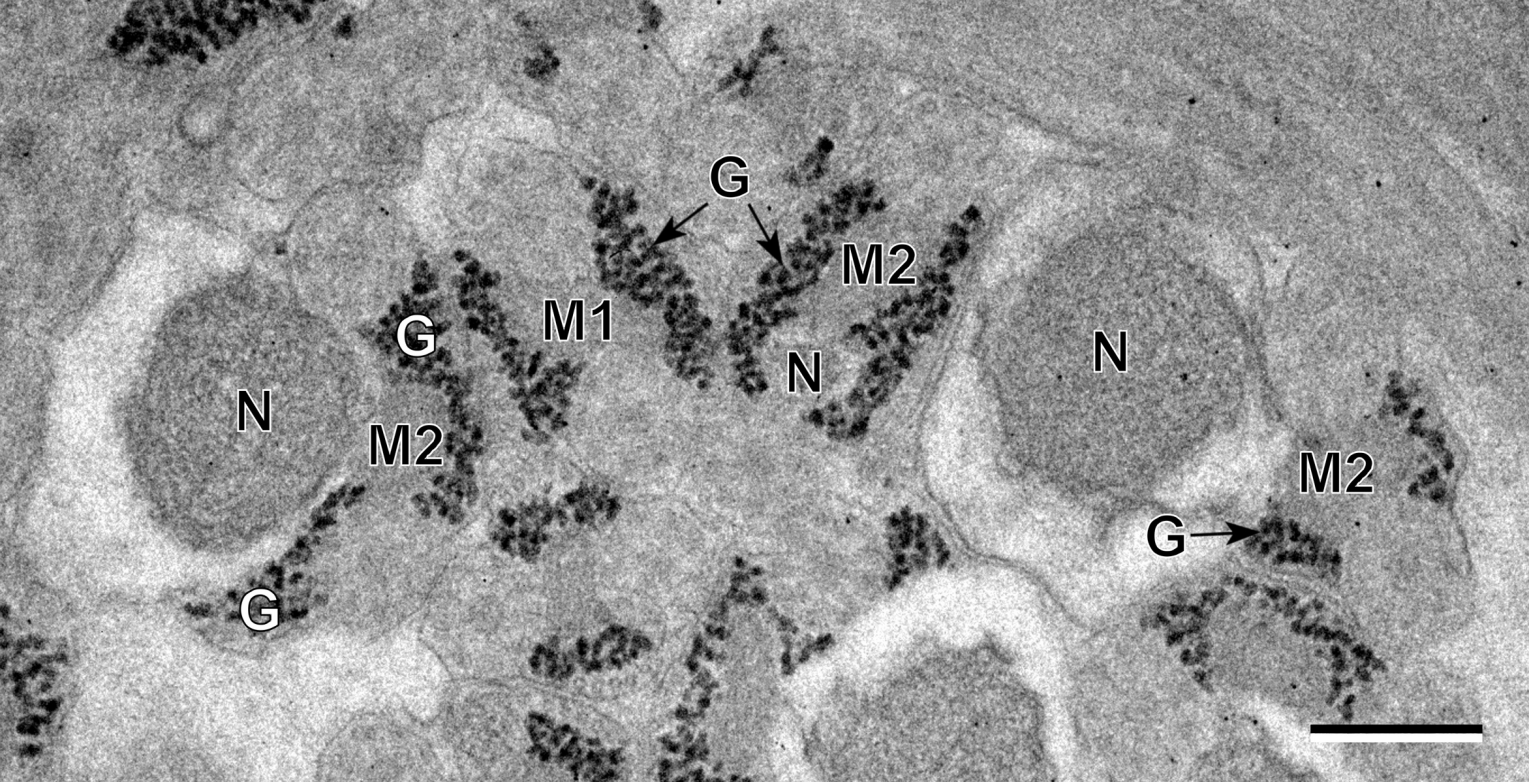
Evidence of the glycogenic nature of electron-dense granules by means of the test of Thiéry in the case of *Pleurogenoides medians*. G, granules of glycogen; M1, first mitochondrion; M2, second mitochondrion; N, nucleus. Scale in μm: 0.3.

**Figure 8.**
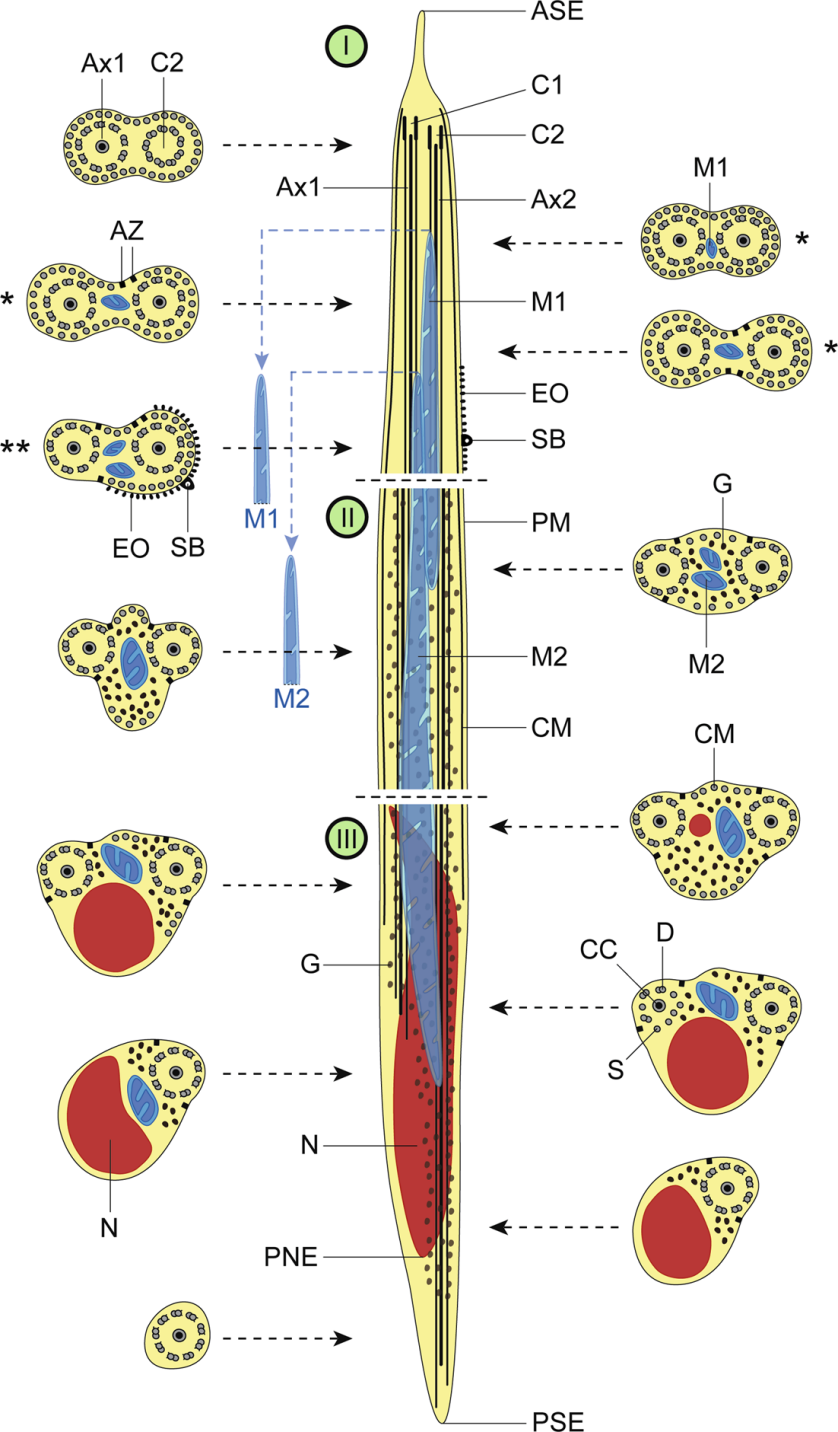
Schematic reconstruction of the mature spermatozoon for the family Pleurogenidae. The sperm cell is organized in three different regions: Region I or anterior part, Region II or middle part and Region III or posterior part. The blue discontinuous lines linked to the first and second mitochondria indicate the more posterior location of the two mitochondria in the spermatozoon of *Prosotocus confusus*. Consequently, in *P. confusus* cross-sections marked with * do not present mitochondrion and the section marked with ** only exhibits the first mitochondrion. ASE, anterior spermatozoon extremity; Ax1, first axoneme; Ax2, second axoneme; AZ, attachment zones; C1, centriole of the first axoneme; C2, centriole of the second axoneme; CC, central core; CM, cortical microtubules; D, doublets; EO, external ornamentation; G, granules of glycogen; M1, first mitochondrion; M2, second mitochondrion; N, nucleus; PM, plasma membrane; PNE, posterior nuclear extremity; PSE, posterior spermatozoon extremity; S, singlets; SB, spine-like bodies.

The observation of numerous cross- and longitudinal sections allows us to distinguish three different regions from the anterior to the posterior extremity of mature spermatozoa of the three studied pleurogenids (*Pleurogenes claviger*, *Pleurogenoides medians* and *Prosotocus confusus*). Each of these three regions exhibits distinctive ultrastructural characteristics shown in Figures 1–8. The usual characters found in the mature spermatozoon of most digeneans are also present in the male gamete of these pleurogenids, i.e. two axonemes of the 9+“1” trepaxonematan pattern, external ornamentation of the plasma membrane, nucleus, mitochondrion, parallel cortical microtubules and granules of glycogen. Other particular features as the morphology of both extremities and the presence of spine-like bodies also characterize the spermatozoon of these species.

- Anterior part or Region I (Figures 1a–g, 3a–j, 5a–h and 8I) corresponds to the anterior extremity of the spermatozoon. The anterior part of this region is filiform, devoid of axonemes and moderately electron-dense (Figures 1a, 3a–d, 5a and 8I). The axonemes of the 9+“1” trepaxonematan pattern are slightly longitudinally displaced (Figures 3c, d, 5b–d, 8I). Both axonemes are surrounded by a continuous and submembranous layer of parallel cortical microtubules (Figures 1c, 3e, 5e and 8I). Posteriorly, the appearance of two and later four attachment zones is observed and thus, parallel cortical microtubules become arranged into two fields (Figures 1d, 3f, g, 5f and 8I). The first mitochondrion is observed in this region (Figures 1d, e, g, 3e–j, 5g, h and 8I). However, there are slight differences in the location of the first mitochondrion within Region I of the studied pleurogenids. In fact, in *P. claviger* and *P. medians* the first mitochondrion appears in the anterior areas of Region I (Figures 1d and 3e–g) while in *P. confusus* it is observed in the ornamented area (Figure 5g, h). Other characteristics of Region I consist in the presence of spine-like bodies and external ornamentation of the plasma membrane in the posterior area of this region (Figures 1e–g, 3i, j, 5g, h and 8I). These external ornamentations are observed associated with cortical microtubules (Figures 1e, g, 3i, j and 5g, h). In the case of *P. claviger*, the spine-like body periodicity was evaluated as 0.5–0.6 μm from a longitudinal section; this was not possible for the two other species (Figure 1f). Finally, as for the first mitochondrion, there are also slight differences concerning the second mitochondrion of the three studied species. In *P. claviger* and *P. medians* it is observed simultaneously with the first mitochondrion in the ornamented area (Figures 1e, g and 3i, j). Contrarily, in the spermatozoon of *P. confusus* the second mitochondrion appears in Region II (Figure 5j–l).
- Middle part or Region II (Figures 1h, 3k, 5k, l and 8II) is mainly characterized by the appearance of electron-dense granules, the presence of the second mitochondrion and the end of the first mitochondrion.
- Posterior part or Region III (Figures 2a–h, 3l, 4a–h, 5m, n, 6a–h and 8III) corresponds to the nuclear region of the spermatozoon. Consecutive cross-sections show: (a) the increasing size of the nucleus (Figures 2a, b, 3l, 4a, and 5m, n); (b) the progressive reduction of cortical microtubules until their disappearance (Figures 2b–d, 4a, b, 6a, b and 8III); (c) the disorganization of the first axoneme (Figures 2e, 4c, 6c and 8III); (d) the end of second mitochondrion (Figures 2e, f, 4d, e, 5d, e and 8III) and (e) the reduction of nucleus size until its end (Figures 2f, g, 4e–g and 6e–g). Consequently, the posterior spermatozoon extremity of these species only exhibits the second axoneme (Figures 2g, h, 4h and 6g). Figure 4a clearly shows the transition of characters in the posterior spermatozoon extremity of *P. medians*, and Figure 6h shows the presence of some granules of glycogen in the posterior spermatozoon tip of *P. confusus*.

The glycogenic nature of the granular material observed in Regions II and III of the mature spermatozoa of the three studied pleurogenids was evidenced by the test of Thiéry (Figure 7).

## Discussion

The mature spermatozoa of *Pleurogenes claviger*, *Pleurogenoides medians* and *Prosotocus confusus* exhibit the same ultrastructural characteristics as most digeneans described so far: two axonemes of the Ehlers’ [20] 9+“1” pattern of trepaxonematan Platyhelminthes, nucleus, mitochondria, granules of glycogen, external ornamentation and two bundles of parallel cortical microtubules. Other additional aspects include the presence of spine-like bodies in the ornamented area of the sperm cell and the continuous submembranous layer of cortical microtubules in the anterior extremity of the spermatozoon. Moreover, the anterior sperm tip is sharp and filiform, and the posterior extremity contains only one of the axonemes.

### Anterior spermatozoon extremity

The anterior tip of the mature spermatozoa of the three pleurogenids is sharp and filiform and the two centrioles appear almost simultaneously at the base of this thin extremity. The two axonemes of different lengths present in the sperm cell of pleurogenids are slightly longitudinally displaced. They exhibit the classical pattern present in the flagella and axonemes of all the Trepaxonemata [20]. Only schistosomes and some didymozoids show a different pattern in axonemal structure [37, 40, 41]. In the pleurogenids studied in the present work, a continuous and submembranous layer of parallel cortical microtubules initially surrounds the axonemes. Such organization of cortical microtubules has been observed in several species such as *Crepidostomum metoecus* (Allocreadiidae) [76], *Brachycoelium salamandrae* (Brachycoeliidae) [12], *Deropristis inflata* (Deropristidae) [24], *Dicrocoelium dendriticum* and *D. hospes* (Dicrocoeliidae) [1, 19], *Euryhelmis squamula* (Heterophyidae) [6], *Hypocreadium caputvadum* (Lepocreadiidae) [44], *Rubenstrema exasperatum* (Omphalometridae) [7], *Postorchigenes gymnesicus* (Phaneropsolidae) [25], *Enodiotrema reductum* and *Plagiorchis elegans* (Plagiorchiidae) [62, 64], *Mediogonimus jourdanei* (Prosthogonimidae) [8], *Troglotrema acutum* (Troglotrematidae) [52] or *Diphterostomum brusinae* (Zoogonidae) [49]. This organization of cortical microtubules without attachment zones indicates the absence of fusion between the free flagella and the median cytoplasmic process in the most basal or proximal area of the differentiation zone during spermiogenesis. This fact was demonstrated for several species and is clearly illustrated in the case of *R. exasperatum* or *T. acutum* [7, 52].

### Spine-like bodies

Spine-like bodies consist in prominent electron-dense structures that seem to contain a spherical vesicle and are usually located in the ornamented area of the spermatozoon. These elements were described only in the Digenea. Miquel *et al.* [52] discussed the similarities and differences between spine-like bodies of digeneans and crested bodies present in most cestodes. In cross-sections spine-like bodies and crested bodies resemble each other. However, spine-like bodies are isolated elements while crested bodies consist in one or more helical cords that surround the spermatozoon.

Spine-like bodies were described for the first time in the opecoelid *Opecoeloides furcatus* [53]. However, they were probably misinterpreted as artefacts of fixation or omitted in previous studies, as in *Paragonimus ohirai* [68], which clearly shows spine-like bodies (see Figure 7 in [68]). To our knowledge, spine-like bodies have been observed in the allocreadiid *C. metoecus* [76], the apocreadiid *Neoapocreadium chabaudi* [45], the brachycoeliid *B. salamandrae* [12], the cryptogonimids *Adlardia novaecaledoniae*, *Anisocoelium capitellatum* and *Aphallus tubarium* [23, 69, 85], the dicrocoeliid *D. hospes* [1], the diplodiscid *Diplodiscus subclavatus* [14], the fasciolids *Fasciola hepatica* and *F. gigantica* [57, 59], the gastrothylacid *Carmyerius endopapillatus* [82], the gyliauchenids *Gyliauchen* sp. and *Robphildollfusium fractum* [13, 72], the mesometrids *Centroderma spinosissima*, *Elstia stossichianum* and *Wardula capitellata* [9–11], the notocotylid *Notocotylus neyrai* [58], the omphalometrid *R. exasperatum* [7], the opecoelids *Helicometra epinepheli*, *Nicolla testiobliquum*, *N. wisniewskii*, *O. furcatus* and *Poracanthium furcatum* [47, 53, 73, 74, 77], the opistholebetid *Heterolebes maculosus* [70], the paragonimid *P. ohirai* [68], the paramphistomids *Cotylophoron cotylophorum* and *Paramphistomum microbothrium* [80, 81], the plagiorchids *E. reductum* and *P. elegans* [62, 64], the pronocephalids *Cricocephalus albus* and *Pleurogonius truncatus* [60, 63], the prosthogonimid *M. jourdanei* [8], the troglotrematid *T. acutum* [52] and the pleurogenids *P. claviger*, *P. medians* and *P. confusus* (present study).

A periodicity in the presence of spine-like bodies was described in some species. For example, periodicity was estimated as 1 μm in *F. gigantica*, *O. furcatus* and *N. testiobliquum* [53, 57, 74], 0.7 μm in *P. furcatum* [47] or 0.6 μm in *N. wisniewskii* [77]. Their distribution is irregular in numerous species, e.g. *R. exasperatum* or *W. capitellata* [7, 11]. In the case of *P. claviger* the location of spine-like bodies seems to follow a periodicity of 0.5–0.6 μm. Nevertheless, in most of the previously cited species spine-like body periodicity is not evident because of the difficulty in observing longitudinal sections containing successive spine-like bodies.

### External ornamentation

The external ornamentation is another structure described in numerous digeneans such as those included in the above-mentioned families and in other families as well: Aephnidiogenidae with the species *Holorchis micracanthum* [4], Brachylaimidae with *Scaphiostomum palaearcticum* [56], Bucephalidae with *Bucephaloides gracilescens* (probably *Prosorhynchoides borealis* according to Bartoli *et al.* [16]) and *Pseudorhipidocotyle elopichthys* [21, 84], Cladorchiidae with *Basidiodiscus ectorchis* and *Sandonia sudanensis* [2], Deropristidae with *D. inflata* [24], Didymozoidae with *Gonapodasmius* sp. [38], Echinostomatidae with *Echinostoma caproni* [30], Faustulidae with *Pronoprymna ventricosa* [75], Haematoloechidae with *Haematoloechus* sp. [39], Haploporidae with *Saccocoelioides godoyi* [15], Hemiuridae with *Lecithocladium excisum* and *Parahemiurus merus* [54, 55], Heterophyidae with *E. squamula* [6], Lecithasteridae with *Aponurus laguncula* [71], Lepocreadiidae with *H. caputvadum* [44], Microphallidae with *Microphallus primas* [18], Monorchiidae with *Monorchis parvus* [48], Phaneropsolidae with *P. gymnesicus* [25], Sclerodistomidae with *Prosorchis palinurichthi* [61] and Zoogonidae with *D. brusinae* [49]. The present study enlarges the number of digenean species and families that exhibit external ornamentation in the spermatozoon. In the digenean sperm cell, the external ornamentation is present in different locations. According to Quilichini *et al.* [72] there are three types of anterior spermatozoon regions depending on this character: (i) type 1 presents external ornamentation in the anterior extremity of the spermatozoon, (ii) type 2 presents external ornamentation at a more posterior level, usually in the mitochondrial region, and (iii) type 3 lacks external ornamentation. According to this classification, the pleurogenids studied in the present work are included in the second type.

Thus, external ornamentation and/or spine-like bodies are present in the spermatozoon of the great majority of digeneans studied until now. The role of these elements remains unknown, but they probably constitute important structures in the process of fertilization considering their location in the male gamete. In fact, the external elements associated to the plasma membrane are always anterior to the nuclear region and in most cases they are located in anterior areas of the sperm cell. During fertilization in digeneans but also in monogeneans, the anterior part of sperm cell coils around the oocyte, penetrating it by lateral fusion [42, 43]. Moreover, several authors hypothesized that the external ornamentation participates in the fusion of the spermatozoon and oocyte membranes during the fertilization process [39]. In addition to their aberrant morphology, schistosomes lack these anterior structures [37]. Certain cestodes also exhibit anterior external structures in the male gamete such as the helical crested bodies that probably also play an important role during fertilization [34, 36, 46].

### Posterior spermatozoon extremity

The posterior tip of digenean spermatozoa is morphologically variable. Quilichini *et al.* [70] distinguished three types of posterior parts of the spermatozoon (the Opecoelidean type, the Fasciolidean type and the Cryptogonimidean type). These types are characterized by the sequence of characters towards the posterior spermatozoon tip. The type 1 or Opecoelidean type is characterized by the sequence “axoneme, nucleus and cortical microtubules”. The type 2 or Fasciolidean type presents the sequence “cortical microtubules, axoneme and nucleus”. Finally, the type 3 or Cryptogonimidean type follows the sequence “cortical microtubules, nucleus and axoneme”. According to these authors, there is a possibility of a fourth group characterized by a different sequence: posterior extremity of the first axoneme, posterior extremity of cortical microtubules and posterior extremity of the second axoneme. This group would be represented by the brachylaimid *S. palaearcticum* [56], the deropristid *D. inflata* [24] and the lecithasterid *A. laguncula* [71]. However, *A. laguncula* does not coincide exactly with this pattern because cortical microtubules end before the axonemes do. Moreover, this species exhibits a unique morphological pattern, in which the mitochondrion reaches the posterior spermatozoon extremity [71]. In our study, each of the three Pleurogenidae studied species exhibits the second axoneme in its posterior spermatozoon tip, an arrangement which corresponds to the Cryptogonimidean type or type 3 posterior spermatozoon extremity. Therefore, according to other authors [7] in order to minimize the existing variations in these sequences, it would be more interesting to consider only the terminal characteristic rather than the succession of characters towards the posterior spermatozoon extremity.

### Perspectives

In the future, the present study can contribute to a better knowledge of relationships between the Digenea and particularly between the Microphalloidea. The taxonomic status of pleurogenids is controversial. This group of digeneans was initially established as a subfamily included in the Brachycoeliidae or in the Lecithodendriidae depending on the authors [84]. More recently, it was raised to family rank, although some taxonomists do not accept this familial status. All these controversies demonstrate that the classical morphological characters alone cannot resolve the existing systematic confusion in numerous taxa such as the pleurogenids [65, 87, 88]. Thus, as numerous authors advocate, a multidisciplinary approach to the systematics and phylogeny of Platyhelminthes is crucial, for example by integrating molecular and ultrastructural tools [27–29]. The molecular analysis of Digenea by Olson *et al.* [65] shows the Microphalloidea as composed of two clades: the first constituted by the Pachypsolidae, the Renicolidae and the Eucotylidae, and the second formed by the Zoogonidae+Faustulidae, the Lecithodendriidae, the Microphallidae, the Pleurogenidae and the Prosthogonimidae. Unfortunately, there are no spermatological data on the families nested in the first clade. On the other hand, in another molecular study, Tkach *et al.* [87] considered that the Microphalloidea were composed of three clades corresponding to diverse families, the lecithodendriids, the microphallids and the prosthogonimids+pleurogenids. Moreover, these authors established several subclades within the Pleurogenidae. The analysis of the available spermatological data shown in Table 1 for microphalloideans emphasizes the necessity to complete additional studies on the spermatozoon of other families, thus allowing for comparisons with molecular data. Presently, sperm ultrastructural data confirm the proximity of pleurogenids and prosthogonimids as evidenced in molecular analysis [87]. Unlike the remaining families, in pleurogenids and prosthogonimids the two axonemes and spine-like bodies are present in the anterior spermatozoon extremity (see Table 1). Moreover, data concerning ultrastructural studies of the spermatozoon in some microphalloideans [18, 26] are not included in Table 1 because they do not show the complete ultrastructural organization of the male gamete and they also contain numerous misinterpretations. This emphasizes the need for a thorough analysis of the spermatozoon in order to (1) avoid missing data on any of the characters and (2) allow for comparisons among all available studies.

**Table 1.** Spermatological characteristics of the Microphalloidea.

Families and species	ASE	EO	LE	SB	M	PSE
Faustulidae						
*Pronoprymna ventricosa* [75]	1Ax	+	−	−	1	1Ax
Pleurogenidae						
*Pleurogenes claviger* [present study]	2Ax	+	−	+	2	1Ax
*Pleurogenoides medians* [present study]	2Ax	+	−	+	2	1Ax
*Prosotocus confusus* [present study]	2Ax	+	−	+	2	1Ax
Phaneropsolidae						
*Postorchigenes gymnesicus* [25]	1Ax?	+	−	−	2	1Ax
Prosthogonimidae						
*Mediogonimus jourdanei* [8]	2Ax	+	−	+	1	1Ax
Zoogonidae						
*Diphterostomum brusinae* [49]	1Ax	+	−	−	1	N

ASE, anterior spermatozoon extremity; Ax, axoneme; EO, external ornamentation; LE, lateral expansion; M, number of mitochondria; N, nucleus; PSE, posterior spermatozoon extremity; SB, spine-like bodies; +/-, presence/absence of considered character.
